# Autologous Bone Marrow Transplantation in Multiple Sclerosis: Biomarker Relevance for Patient Recruitment and Follow up

**DOI:** 10.4172/2155-9899.1000455

**Published:** 2016-09-20

**Authors:** Ana C. Londoño, Carlos A. Mora

**Affiliations:** 1 Instituto Neurológico de Colombia-INDEC (A.C.L.), Medellin, Colombia; 2 Department of Neurology (C.A.M.), Georgetown Multiple Sclerosis Center, MedStar Georgetown University Hospital, Washington, DC, USA

**Keywords:** Autoimmune diseases, Bone marrow transplant, Cerebrospinal fluid, Hematologic, Multiple sclerosis, MRI, Stem cell

## Abstract

**Background:**

Despite the current availability of disease modifying therapies for the treatment of multiple sclerosis, there are still patients who suffer from severe neurological dysfunction in the relapsing-remitting or early progressive forms of the disease. For these patients autologous hematopoietic stem cell transplant offers an important therapeutic solution to prevent progression to irreversible disability. In spite of multiple studies in the last two decades, patient inclusion criteria, protocols for peripheral blood stem cell mobilization and bone marrow cell conditioning and methodology of follow up for autologous hematopoietic stem cell transplant in multiple sclerosis have not been strictly unified.

**Methods:**

We reviewed five recent clinical studies that confirmed the positive outcome of transplant in spite of disclosing significant differences in methodology of enrollment including patient disease subtypes, disease duration range, disability, regimens of peripheral blood stem cell mobilization and bone marrow cell conditioning, scheduling of imaging studies after transplant, and absence of laboratory biomarkers consistently applied to these studies.

**Results:**

Therapy with autologous hematopoietic stem cell transplant has shown best results among young individuals with severe relapsing-remitting or early progressive disease through its ability to maintain no evidence of disease activity status in a significantly higher proportion of patients after transplant in comparison to patients treated with disease modifying therapies. Important cross-sectional differences in the reviewed studies were found.

**Conclusion:**

A specific and careful selection of biomarkers, based on the current physiopathological mechanisms known to result in multiple sclerosis, will contribute to a better and earlier patient selection for autologous hematopoietic stem cell transplant and follow up process. An objective and measurable response could be obtained with the determination of biomarkers at the onset of treatment and after follow-up on reconstitution of the immune response. The application of such parameters could also help further our understanding of pathogenesis of the disease.

## Introduction

A main challenge frequently observed in the clinical practice of multiple sclerosis (MS) is the treatment of young patients presenting with frequent episodes of severe, sometimes catastrophic, impairment of neurological functions that characterize the relapsing-remitting form of the disease (RRMS) when it is refractory to the current disease modifying agents. A therapeutic alternative especially recommended for such individuals has been the autologous hematopoietic stem cell transplantation (AHSCT). The goal of therapy with AHSCT is the induction of an intense depletion of T-cells, resulting in the eradication of autoreactive cells, leading to a resetting of patient’s immune system and availability of a new and diverse T-cell repertoire generated by thymic output [1,2]. This has been possible through the infusion of autologous CD34^+^ cells previously collected from the bone marrow or by leukapheresis from peripheral blood after conditioning regimen (which may offer high, intermediate or low myeloablative effect) [2,3]. Thus, reduction of encephalitogenic Th17 cells and transient increment of regulatory FoxP3^+^ T-cells and CD56high natural killer (NK) cells may help arrest disease progression and possibly recover lost neurological function [3]. Contrary to a myeloablative conditioning regimen, a non-myeloablative regimen may prevent bone marrow suppression and may improve safety and tolerability [4]. Since 1995, AHSCT has been an alternative in the treatment of patients with autoimmune diseases [5]. In 1997, Fassas et al. published the first study exploring the feasibility of AHSCT in the treatment of progressive MS [6]. Although AHSCT has been a therapeutic alternative for hundreds of MS patients for more than 15 years, its effect on the peripheral immune system and central nervous system (CNS) pathology is still a matter of investigation.

## Peripheral immunity and CNS pathology

The information available on the effect of AHSCT on CNS pathology mainly stems from studies in animal models that most closely resemble human disease. In a review by Cartier et al. the following immunological events were described in the CNS of animal models of myeloablative transplant: 1) replacement of CNS perivascular macrophages and microglia by bone marrow derived cells; 2) reconstitution process thanks to hematopoietic stem cells containing a sub-population of cells able to replace the precursors of intraparenchymal microglia; 3) a myeloablative transplant protocol that includes busulfan may facilitate the exchange of microglia with donor cells since it depletes the endogenous microglia and favors the establishment of new cells; 4) blood-derived monocytes seem to have the ability to infiltrate and settle down in brain areas depleted of microglia; and 5) a disruption in the blood brain barrier (BBB) is required for the penetration of myeloid progenitors in the brain parenchyma and their corresponding differentiation in microglia-like cells [7]. In addition, in lethally irradiated mice, upon bone marrow transplantation genetically modified hematopoietic cells differentiated into CNS microglia and by four months after transplantation up to a quarter of the regional microglia were donor derived [8].

Using a mouse model of bone marrow transplant (BMT) in experimental autoimmune encephalomyelitis (EAE) Cassiani-Ingoni et al. were able to demonstrate that transplanted cells produce a complete reconstitution of the peripheral immune compartment but the exchange of cells between blood and brain was predominantly confined to the sites of lesion. They also found that BMT may block or delay EAE progression when it is conducted in an early stage while no modification of the clinical course happens if it is done at a later stage. In animals with disease progression, histology showed reduced lymphocytic infiltration with prominent activation of endogenous macrophage/microglia. In addition, endogenous Olig2^+^ glial progenitor cells were found to maturate into reactive astrocytes depending upon the type of lesions, stage of disease or repair process [9]. In a mouse model of Alzheimer disease for determination of the role of genotype apolipoprotein E (APOE) [which modulates CNS innate immune function in cell cultures] in disease progression, Yang et al. compared outcome after myeloablative BMT from APOE3/3 and APOE4/4 donors. Eight months later, there was no difference in the proportion of T and B lymphocytes and neutrophils in blood, except for a higher number of monocyte/macrophage CD11b linage cells in the APOE3/3 receptors. Also, replacement of the microglia in the cerebral cortex and hippocampus was 1/3 in BMT from APOE4/4 compared with almost 1/2 in BMT from APOE3/3 in a relatively anti-inflammatory environment (shown by the reduced expression of TNF-α and macrophage migration inhibitory factor and by the elevation of the expression of IL-10 in the APOE3/3 BMT receptors compared with the APOE4/4 BMT receptors) [10]. Another contribution of the mouse model of EAE is that it has demonstrated that non-myeloablative and less toxic conditioning generates autoantigen-encoding bone marrow that promotes tolerance, with low levels of chimerism. Besides, it prevents relapses and also reverses established disease [11,12].

Abrahamson et al. were able to assess the immune cellular response and reconstitution of immune adaptive system two years after nonmyeloablative AHSCT in MS patients disclosing a favorable balance with expansion of regulatory T-cells in peripheral blood. A high percentage of these cells were of thymic origin and showed significant depletion of mucosal-associated invariant T-cells (MAIT) of intestinal origin, corresponding to CD161^high^ CD8^+^ T cells, which are known producers of IFN-γ, TNF-α and IL-17 [4]. Furthermore, MAIT cells expressing CCR6, a receptor involved in the transmigration of T-cells into the CNS and in the induction of EAE, have the exclusive ability to enter the CNS and their presence has been confirmed in active white matter lesions of CNS specimens from patients with MS [4]. The MS patients who had initially presented with significant proliferation of MAIT cells, had almost no detectable presence of these cells in peripheral blood following non-myeloablative AHSCT treatment [4]. The Canadian Collaborative MS/BMT study on patients who underwent high myeloablative regimen with subsequent clinical and cell reconstitution follow up for two years demonstrated re-emergence and in-vivo expansion of CNS-auto reactive T-cells [13]. The T-cell repertoire, however, exhibited a significant reduction of Th17 and Th1/17 responses, rather than Th1 responses. The corresponding chemokine network was modified following AHSCT and CD4 cells took longer to reach pre-treatment level even after 2 years post-transplant, while the CD8 cells returned promptly to their baseline resulting in a sustained inversion of the CD4/CD8 ratio. Thymopoiesis was restored in MS at the pre-treatment level allowing normal generation and release of the recent thymic emigrant cells [13]. Using high-throughput deep TCRβ chain sequencing, changes in the T cell repertoire post-AHSCT included the presence of a new repertoire of CD4^+^ cells and the lack of effective removal of CD8^+^ T cells, the reconstitution of which was secondary to clonal expansion of cells present before transplant. In fact, patients who responded to treatment had more diversity in their T cell repertoire early during the reconstitution process [14]. Reconstitution of B cells in AHSCT is characterized by a slow increment of CD19^+^ lymphocytes being able to reach a normal value six months after transplant [2]. In allogeneic hematopoietic stem cell transplantation (HSCT) B cells were found to be rare in peripheral blood during the first months after transplant reaching close to normal levels within 6–12 months; memory B cells expressing CD27 were found at subnormal levels during the first two years after transplantation; and recipient-derived B cells could be reduced right after transplantation, especially after following a reduced intensity conditioning regimen [15]. The reconstitution of antibody subclasses was also seen during the first year with the sequential presence of IgM, IgG1/IgG3, IgG2/IgG4 and IgA antibodies. In recipients of allogeneic HSCT who developed graft-versus-host disease (GVHD) many phenotypic changes were encountered in B cells with delayed B cell reconstitution [15]. In a study on B cell exchange across the BBB in MS, Von Büdingen et al. found that the migration of B cells is limited, or inexistent, in normal conditions but magnified in the process of several diseases including MS. B cell exchange can be bidirectional and raise the possibility that B cell-mediated autoimmunity can originate and persist on both sides of the BBB in MS. According to the degree of inflammation in MS, migrant cells to the CNS could be susceptible to expansion and/or clonal diversification [16].

## Patient selection

Inclusion criteria for enrollment in clinical trials with AHSCT have typically considered the expanded disability status scale (EDSS) scores, the annualized relapse rate (ARR) and the brain magnetic resonance imaging (MRI) changes as the hallmark biomarkers that define initiation of therapy and determination of success or failure during follow up. These classic tools, however, may not fully correlate with the immunological cascade of events that characterize the pathophysiology of MS since they help identify major changes in stages of inflammation and neurodegeneration but may fail to detect subclinical and progressive changes at the biochemical and cellular levels. For this reason, patient selection for AHSCT based on the above-mentioned markers, may lead to enrollment of patients in advanced stages of the disease with higher EDSS scores reflecting an advanced neurodegenerative process and a poor chance of functional recovery. It could also lead to the exclusion of patients at higher risk for aggressive disease and catastrophic course i.e., the potential beneficiaries of AHSCT. Besides, this methodology has not been effective in predicting early disease reactivation.

## Recent studies

Five recent clinical studies have confirmed the positive outcome of AHSCT especially among groups of patients with mean age range 34–38 who presented with rapid progression; with EDSS scores between 1.5 and 8.0; with radiological evidence of high inflammatory activity by MRI of the neuroaxis and with disease progression ranging from 1.3 to 11.2 years respectively [17–21]. Comparison of data that stemmed from these five studies discloses significant differences in methodology including: 1) patient enrollment characteristics such as disease subtypes, disease duration range and EDSS; 2) heterogeneous regimens of peripheral blood stem cell mobilization and bone marrow cell conditioning, 3) diverse scheduling of imaging studies after transplant, and 4) absence of laboratory biomarkers consistently applied to these studies (Table 1). None of these five studies reported analysis of body fluids, including cerebrospinal fluid (CSF), as point of reference during baseline or follow-up but for the peripheral blood lymphocytic population analysis described by Darlington et al. [13] (as part of the study recently reported by Atkins et al. [21]).

In separate reports, Curro et al. [2] and Radaelli et al. [3] recently reviewed a combination of 29 series on AHSCT in MS published in the medical literature from 1997 to 2015. In these series, patients presented with different subtypes of disease, wide ranges of EDSS scores and duration of disease, and differences in the methods of cell mobilization and conditioning at the time of transplant. In addition, patient selection and follow-up were especially based on clinical and radiological criteria without the inclusion of determination of biomarkers in body fluids. These studies led to the conclusion that the best response to AHSCT was seen in young individuals with the RRMS subtype, short clinical course and rapid progression of disease. Besides, the BEAM/ATG (combination of carmustine, etoposide, Ara-c and melphalan with rabbit anti-thymocyte globulin) protocol showed to be safer than protocols including busulfan during mobilization [2,3].

## Biomarkers

The study of body fluids including fresh blood, serum and CSF, has allowed classifying biomarkers for different stages of MS, as well as prognosis [22]. The application of a method that would give significant priority to the use of specific biomarkers of inflammation and degeneration, as predictors of disease progression and response to therapy, will result in better patient selection for AHSCT thus improving prognosis. Patients who carry the HLA-DRB1*15:01 allele have shown positive correlation with oligoclonal bands (OCB) in CSF, early disease onset, risk of cognitive decline, presence of bigger white matter lesions, more advanced brain atrophy, higher lesion load on MRI, and higher concentration of matrix metalloproteinase-9 (MMP-9) in CSF [23,24]. T-cells and activated monocytes and macrophages have the ability to produce a variety of matrix metalloproteinases, including MMP-9, which cause degradation of the extracellular matrix and facilitate migration of leucocytes through the basement membranes [25]. The proteolytic activity of MMP-9 influences the permeability of the BBB, hence high levels of MMP-9 correlate with disease activity on gadolinium-enhanced brain MRI [26,27]. Ljubisavljevic et al. reported increased plasma levels of matrix metalloproteinase-3 (MMP-3) and MMP-9 among patients with clinically isolated syndrome (CIS) and RRMS during relapses in comparison to control group. Patients also showed a positive correlation between plasma values of MMP-3 and MMP-9 and increment of neurological disability; number of T2wi white matter lesions; volume of gadolinium enhancing lesions and permeability index of the BBB [28]. In addition, the combined determination of CXCL13, IL-8 and IL12-p40 in CSF establishes the presence of active inflammation in CNS [29]. Also, in MS patients with IgM OCB against myelin lipids, the course of disease will be characterized by earlier occurrence and increased frequency of relapses, earlier progression toward disability, and increment of percentage of CD5^+^ B-cell population in peripheral blood and, remarkably, in CSF [30,31]. Two additional CSF biomarkers include soluble CD27 (sCD27) which is considered an excellent biomarker of T-cell-mediated active intrathecal inflammation in patients with progressive MS [32] and the light chain subunit of neurofilaments (NfL) which is a reliable biomarker of axonal damage [33]. A study on the correlation of oxidative stress and neuroinflammation disclosed high plasma levels of phosphorylated neurofilament heavy chain (pNF-H) in RRMS patients and, to a lesser extent, in CIS patients when compared to control group. In the same study, the oxidative stress marker 8-OHdG (8-hydroxy-2’-deoxyguanosine) was especially increased in plasma of CIS patients [34]. Potential biomarkers for assessment before and after non-myeloablative AHSCT might include presence of MAIT cells as well as Th17 and Th1/17 cells in body fluids [4,13]. In the future, the in-vivo evaluation of microglial activation using PET scan [35] would allow monitoring reactivation and resetting of microglia before and after transplant.

The best outcome of AHSCT therapy has been reported in young patients who presented with RRMS, evidence of active inflammation in the neuroaxis on MRI, short duration of disease, low EDSS score and lack of response to standard therapies [2]. In terms of safety and feasibility, AHSCT treatment related mortality (TRM) -or death due to causes unrelated to the underlying disease but considered as related to transplantation- has dropped from at least 6% in early reports to 1.3%, and progression-free survival has ranged from 47% to 100% [3,6]. It is important to consider that AHSCT therapy may offer, on average, a 3-year period of remission with no need to continue disease modifying therapy in some patients. However, special caution, should be considered in the case of five patients whose EDSS scores at the time of AHSCT ranged from 5.5 to 8.0 and whose autopsy disclosed an apparent persistence of activation of the microglia at the time of death [36]. The period of time between AHSCT and death in this group ranged from 20 days to 1.5 year (median 2 months). These findings could pose the question that whether microglial activation persists after AHSCT or results from production of novel post-transplantation inflammatory cells in the CNS. However, the period between AHSCT and death in this series was relatively short to conclude that AHSCT therapy might fail to prevent demyelination and promote regeneration in MS [37,38].

Based on the available data in animal models of BMT [7] it would be important to evaluate whether or not the integrity of the BBB at the time of transplant has any effect on response to AHSCT in humans. In the Italian multicenter AHSCT study, using an intermediate conditioning regimen, the most significant variable associated with a better clinical outcome was the presence of contrast enhancing lesions in MRI at the time of transplant with a progression free survival (PFS) of 87% at 5 years vs. 46% when MRI failed to show enhancing lesions [39]. This finding supported the presence of better PFS in patients with MRI enhancing lesions in the 15-year follow-up study previously reported by Fassas et al. (44% vs. 10% respectively) [40]. At the same time, it would be important to evaluate whether or not an intense conditioning regimen has an effect on the resetting of microglia in humans with lower risk of developing TRM. This is important considering that busulfan, a drug associated with risk of TRM after AHSCT in humans, has been associated with a high turnover of microglia after transplant in animals [3]. Recently, Atkins et al. reported long term stability in a group of patients who received near-complete immunoablation and AHSCT with modified conditioning including busulfan and CD34 immunomagnetic graft selection. Although 70% patients were free from further progression of disease for 6.7 (3.9–12.7) years, the study reported 2 cases of severe adverse reaction to busulfan including one death [21]. Recent observations on therapy with AHSCT suggest that a low intensity regimen, contrary to an intermediate intensity regime, may fail to produce a prolonged anti-inflammatory effect in MS, as seen by MRI, even though patients may present with similar adverse effects [41]. It would also be important to establish whether the genotype APOE3 or the genotype APOE4 have any effect on the repopulation of microglia after AHSCT, as it has been documented in studies using transgenic mice [10].

A methodic surveillance of the immune cellular function before and during reconstitution following AHSCT would allow identifying those cells that remain out of control and determine the first step in peripheral blood and CNS cell re-activation hence better understanding of pathogenesis. The use of biomarkers for the identification of the key players in pathogenesis i.e., peripheral (T-cells, B-cells, macrophages-monocytes, NK cells and dendritic cells) and central (astrocytes, oligodendrocytes, microglia and neurons) cells and their products play an important role in this process. A careful analysis of the most relevant biomarkers, for purposes of patient selection and follow up after AHSCT therapy, ought to be conducted by a panel of experts in order to determine their function and specificity in the clinical setting including correlation with MRI findings [23]. In near future, the application of systems medicine to clinical practice will help to expedite the integration of basic and clinical science knowledge so that tailored therapeutic interventions –such as AHSCT- become the goal of personalized medicine in patients with MS [42,43]. In a serum proteomics study, eleven proteins were associated with MS disease progression, inflammation, opsonization and complement activation. Although these proteins could help to identify patients in need of a more aggressive treatment they may still require validation [44].

The availability of novel disease modifying therapies, including monoclonal antibodies, may question the use of AHSCT in MS in the future. However, AHSCT prevents relapses, arrests disease progression, and reduces the risk of disability in patients without concomitant use of disease modifying therapies (DMT) [45]. In fact, DMT reduce frequency of relapses but its effect on arresting disease progression has not been totally accomplished [45]. We strengthen the fact that AHSCT still offers an alternative to altering multiple peripheral and CNS immunological factors concomitantly involved in the pathogenesis of MS, which is more difficult to accomplish with the current disease modifying agents. Furthermore, AHSCT could include the use of genetically modified autologous hematopoietic stem cells in the future [7]. By means of genetically modified AHSCT it would be possible to promote immune tolerance toward antigens or a wide variety of molecules, thus permitting the identification and elimination of pathogenic cellular clones or the creation of lymphocytes capable of controlling and suppressing the activity of self-reactive clones [12]. Recent studies in animal models using mesenchymal stem cells transplant or induced pluripotent stem cell (iPSCs) derived precursor cells have shown both neurogenesis, and a therapeutic effect as modifiers of the immune response and promoters of regeneration in CNS diseases including MS [46–48]. Further research is required before recommending transplantation of mesenchymal cells as a forthcoming alternative to AHSCT in patients with MS. An ongoing study entails the assessment of bone marrow-derived cellular therapy in progressive MS (ACTiMuS) and is the first randomized, placebocontrolled trial of non-myeloablative autologous bone marrow-derived stem cell therapy in MS [49].

In conclusion, we believe that expert consensus is needed for the development of a navigational chart in AHSCT therapy for MS patients. This chart, in the form of algorithm or guideline, should steam from unified criteria based on biomarkers of poor prognosis established at time of diagnosis and aimed at patient selection for AHSCT before progression to disability. It should also determine the biomarkers at the baseline and end-point follow-up with better sensitivity and specificity for AHSCT. Besides, it should consider selection of the intensity of conditioning regimen and timing of the transplant. Available data suggest that best results in AHSCT therapy are obtained in young individuals with RRMS entailing an early and high inflammatory component. Sormani et al., sustain that AHSCT has demonstrated ability to maintain no evidence of disease activity (NEDA) status in a significantly higher proportion of patients at 2 years (78–83%) and at 5 years (60–68%) after transplant in comparison to RRMS patients treated with DMTs [50]. In addition, they propose to create a randomized comparative trial to assess the risk-benefit profile of AHSCT in patients with highly active MS not responding to DMTs [50]. We do insist on the application of biomarkers for better AHSCT patient selection and follow-up of progression of disease thus evolving toward the inclusion of a ‘biomarker-based NEDA’ concept in near future.

## Figures and Tables

**Table 1:** Comparison of patient enrollment characteristics, methodology and outcome of five recent studies on AHSCT therapy for multiple sclerosis.

Authors	Mancardi et al.	Nash et al.	Burt et al.	Shevchenko et al.	Atkins et al.
Year	2015	2015	2015	2015	2016
[reference]	[17]	[18]	[19]	[20]	[21]
N# patients/ gender	21 (9 AHSCT, 12 mitoxantrone)/ 14 female	25 (24 at end)/ 17 female	151 (145 at end)/ 85 female	99/ 60 female	24 (21 at end)/ 14 female
Mean age (range)	36 (19–46)	38 (27–53)	36 (18–60)	35 (18–55)	34 (24–45)
Race/ ethnicity	ND	24 white, 1 multiracial	124 white, 9 black,	ND	ND
			7 Asian, 5 Hispanic		
HLA-DRB*15:01 allele	ND	ND	ND	ND	38% heterozygous, 17% homozygous
Median baseline EDSS (range)	6.0 (5.5–6.5)	4.5 (3.0–5.5)	4.0 (3.0–5.5)	3.5 (1.5–8.0)	5.0 (3.0–6.0)
MS type	2 RR, 7 SP	24 RR	123 RR, 28 SP	43 RR, 35 SP, 12 RR,	12 SP
				18 PP, 3 PR	
Disease duration (range), y	10.2 (2–23)	4.9 (0.6–12)	6.6 (0.75–22)	5.0 (0.5–24)	5.8 (1.3–11.2)
Type of study	Phase 2, multicenter, randomized (AHSCT or MTX), 2004–2009	Phase 2, multicenter, prospective, open label, single arm	Open-label, single center study, 2003–2014	Phase 2, single center study, 2005–2011	Phase 2, single arm, 2000–2009
Inclusion criteria	Worsening 1 step in EDSS score in the last year or 0.5 step when EDSS 5.5–6.5	>2 moderate-severe attacks in previous 18 months while on DMT	2 relapses in previous year or 1 relapse and 1 Gd+ lesion in brain MRI	McDonald criteria	EDSS score at least 3.0 within 5 y of disease onset with a cerebellar or pyramidal functional system score of at least 3.0.
					Two disabling attacks in the last year or 3 disabling relapses in the last 2 y.
	EDSS 3.5–6.5	Worsening 1 EDSS step or worsening 0.5 step when EDSS 4.0–5.5	EDSS 1.5–8.0	Normal mental status	Worsening 1 or more EDSS steps in the last 18 months if EDSS was ≤ 5.0, or 0.5 EDSS step if baseline score was >5.5
	MRI: 1 or more Gd^+^ lesions	McDonald diagnostic criteria	McDonald diagnostic criteria	Absence of severe concomitant disease	Paty & Fazekas diagnostic criteria
	Poser diagnostic criteria			Gd^+/−^ enhancing lesions	
				No treatment with IFN or immunosuppressive agents within 3 months before enrollment	
Evidence of inflammatory activity by MRI at baseline	ND	42%	58%	40%	87.5% showed (+) MRI activity during first year before AHSCT
End points	Cumulative # of T2 MRI lesions in the 4 y follow-up, randomized (primary)	Event free survival without death or disease activity	Reversal of progression of disability; changes in NRS, MSFC, SF-36	Long term outcomes on quality of life (clinically observed and patient reported)	Event-free survival without clinical relapse, appearance of a new or Gd^+^ lesion on MRI and without sustained progression of EDSS
	Cumulative # Gd^+^ lesions; relapse rate and disability progression (secondary)	Reduction in Gd^+^ lesions			
Mobilization of autologous human stem cells	Cy (4 g/m^2^)	Prednisone	Cy (2 g/m^2^)	G-CSF (10 mg/Kg)	Cy (4.5 g/m^2^)
	Filgrastim (5 mg/kg)	Filgrastim (16 mg/kg)	Filgrastim (5–10 mg/kg)	Methyl-prednisolone	Filgrastim (10 mg/kg)
Autologous CD34 ex vivo immunomagnetic graft selection	ND	ND	ND	ND	(+) stem cell selection
Conditioning regimen	Intermediate (reduced)-intensity myeloablation (BEAM) + ATG	Intermediate (reduced) -intensity myeloablation (BEAM) + ATG	Low intensity lymphoablative/ immunoablative/ non- myeloablative	Lesser than intermediate intensity myeloablation (mini- BEAM like or carmustine + melphalan)	High-intensity myeloablation (busulfan + Cy + ATG)
			[Cy with either alemtuzumab or ATG + methyl-prednisolone]		
Clinical and MRI follow up design	Baseline, then every 12 months for 4 y	Baseline; 6 and 12 months; annually through the 5th y clinical evaluation (including EDSS, MSFC, MSIS-29)	Baseline; 6 and 12 months; annually through the 5^th^ y	EDSS and QoL assessment at baseline, 3, 6, 9 and 12 months; every 6 months thereafter up to 48 months; then yearly intervals	Baseline; 1, 2, 4, 6, 9, 12, 15, 18, 24, 30, 36 months; then every 6–12 months (EDSS, MRI, MSFC)
	(including EDSS and ARR)	[MRI was also done at 2 months]	(including EDSS, NRS score, MSFC, QoL Short Form 36);	(spinal cord MRI included in follow up)	
			[T2 lesion volume on MRI]	[EDSS and QoL also done at discharge]	
Outcome including relapse (event) free survival (RFS)	79% reduction in number of white matter lesions as compared to MTX arm.	Event-free survival was 78.4% at 3 y based either on clinical or MRI findings	Improvement in disability EDSS scores beyond 1.0 step after 2 y of transplant.	80% had event-free survival at median follow-up of 49 months.	Event-free survival was 69.6% at 3 y.
	100% absence of Gd^+^ lesions during 4 y follow up compared to 56% in MTX arm.				70% had no EDSS progression and a median follow-up of 6.7 y (range 3.9–12.7).
	Reduced ARR as compared to MTX arm.	RFS was 86.3% and PFS was 90.0% at 3 y	RFS was 80% and PFS was 87% at 4 y	47% improved EDSS score by at least 0.5 points on the EDSS and 45% were stable after long-term follow up	100% had absence of MRI activity after transplant.
	No difference in EDSS or progression of disease				35% had sustained improvement in EDSS.
Treatment related mortality (TRM)	0	0	0	0	1
Comments to study	MRI studies during follow up were conducted on an annual basis for four years	Disease duration less than 15 years.	EDSS score did not improve in SPMS or patients with disease duration >10 years.	Disease duration 0.5–24 years	1 patient died (TRM).
	Baseline MRI data was lost in 3 patients	Patients had no acute treatment-related neurologic adverse events.	Six patients were excluded from the outcome analysis.	No statistically significant differences in event survival rate between the conditioning regimes effect.	Two patients censored within 2 y after CCSVI treatment.
		1 patient died of MS progression 2.5 y after AHSCT			
		1 patient died of worsening asthma 3.5 y after AHSCT.	14% patients treated with alemtuzumab developed ITP compared with 3.1% treated with ATG.		Whole brain atrophy slowed to a rate associated with normal aging
					Only study conducting HLA allele determination and stem cell immunomagnetic graft selection.

Columns display data from five studies on AHSCT in MS disclosing significant differences in methodology including: 1) patient enrollment characteristics such as disease subtypes, disease duration range and EDSS; 2) heterogeneous regimens of peripheral blood stem cell mobilization and bone marrow cell conditioning, 3) diverse scheduling of imaging studies after transplant, and 4) absence of laboratory biomarkers consistently applied to these studies. Event-free survival = no evidence of EDSS progression, clinical relapse and MRI event.

Abbreviations: AHSCT: Autologous Hematopoietic Stem Cell Transplant; ARR: Annualized Relapse Rate; ATG : Anti-Thymocyte Globulin; BEAM : Conditioning Regimen Including Carmustine, Etoposide, Ara-c and Melphalan; CCSVI : Chronic Cerebrospinal Venous Insufficiency; Cy : Cyclophosphamide; DMT : Disease Modifying Therapy; EDSS : Expanded Disability Status Scale; G-CSF : Granulocyte Colony Stimulating Factor; ITP : Immune Mediated Thrombocytopenia; MSFC : Multiple Sclerosis Functional Composite; MSIS-29: 29-item Multiple Sclerosis Impact Scale; MTX : Mitoxantrone; ND : No Data; NRS : Neurology Rating Scale; PP Primary Progressive; PFS: Progressive-Free Survival; PR : Progressive Relapsing; QoL : Quality Of Life; RFS : Relapse Free Survival; RR : Relapsing Remitting; SF-36 : Short Term 36 Quality-Of-Life Score; SP: Secondary Progressive; TBI: Total Body Irradiation; TRM: Treatment Related Mortality; WM : White Matter; y : years

## Glossary

AHSCT: Autologous Hematopoietic Stem Cell Transplantation
APOE: Apolipoprotein E alleles
ARR: Annualized Relapse Rate
ATG: Rabbit Anti-thymocyte Globulin
BBB: Blood Brain Barrier
BEAM: Carmustine Etoposide Ara-c Melphalan
BMT: Bone Marrow Transplant
CCR6: Chemokine Receptor CCR6
CIS: Clinically Isolated Syndrome
CXCl13: Soluble factor CXCL13
DMT: Disease Modifying Therapy
EAE: Experimental Autoimmune Encephalomyelitis
EDSS: Expanded Disability Status Scale
G-CSF: Granulocyte-Colony Stimulating Factor
IFN-γ: Interferon gamma
IL-8: Interleukin-8
IL-12: Interleukin-12
IL-17: Interleukin-17
iPSC: induced Pluripotent Stem Cell
MAIT: Mucosal-Associated Invariant T-cells
MMP-3: Matrix Metalloproteinase-3
MMP-9: Matrix Metalloproteinase-9
MS: Multiple Sclerosis
NEDA: No Evidence of Disease Activity
NfL: Light chain subunit of Neurofilaments
NK: Natural Killer cells
OCB: Oligoclonal Bands
PFS: Progression Free Survival
RFS: Relapse Free Survival
RRMS: Relapsing Remitting Multiple Sclerosis
sCD27: Soluble factor CD27
TCRβ: T-cell Receptor β chain
Th1: T-helper cells Type 1
Th17: T-helper cells producing interleukin 17
TNF-α: Tumor Necrosis Factor alpha
TRM: Treatment Related Mortality
